# Supplementary figures and images for: Efficient degradation of various emerging pollutants by wild type and evolved fungal DyP4 peroxidases
Source: PLoS One. 2022 Jan 13;17(1):e0262492. doi: 10.1371/journal.pone.0262492 (PMC8757903; doi:10.1371/journal.pone.0262492)

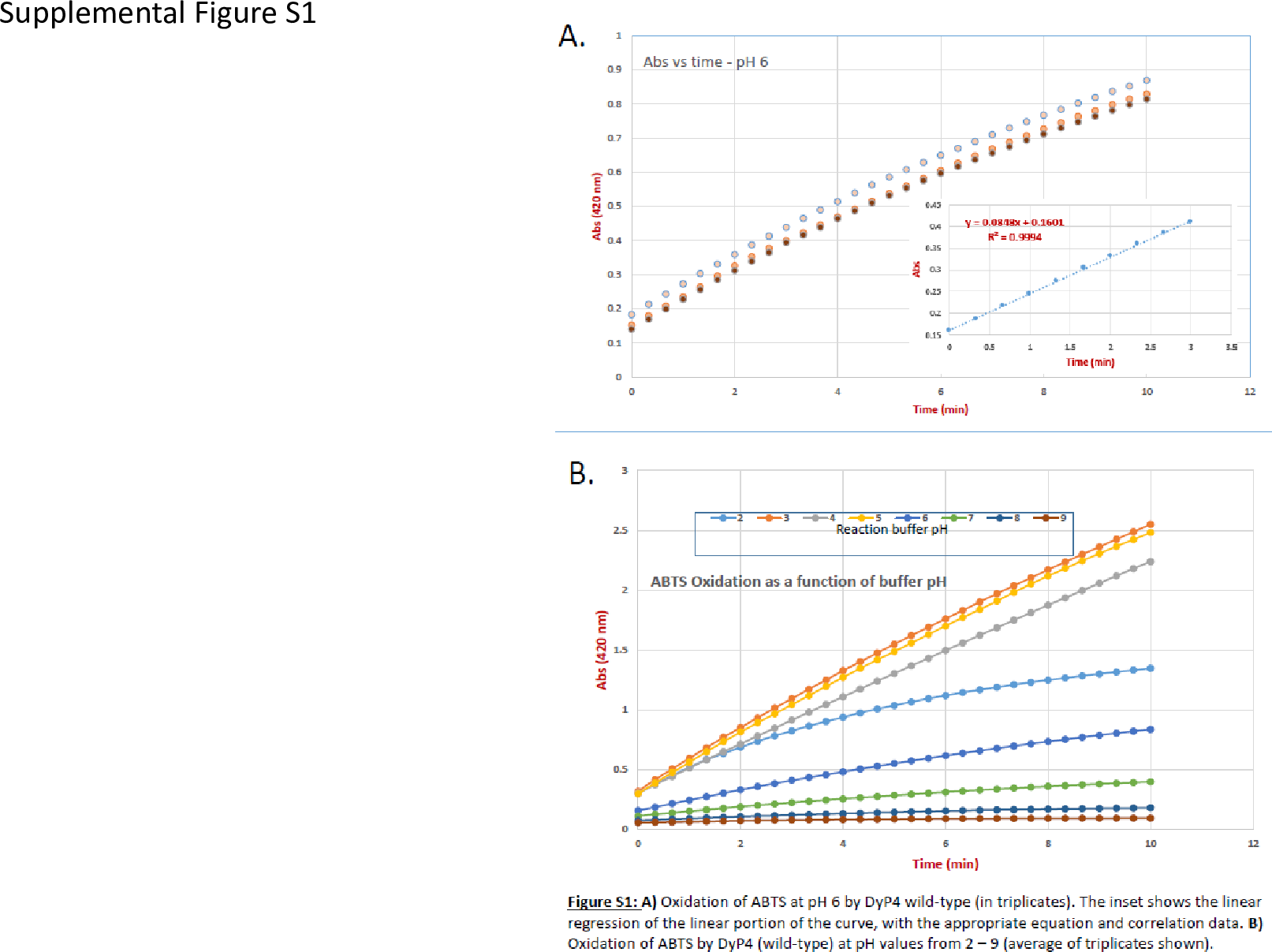

Supplement: S1 Fig — A) Oxidation of ABTS at pH 6 by DyP4 wild-type (in triplicates). The inset shows the linear regression of the linear portion of the curve, with the appropriate equation and correlation data. B) Oxidation of ABTS by DyP4 (wild-type) at pH values from 2–9 (average of triplicates shown). (TIF) [file pone.0262492.s001.tif]
