# Supplementary material for: Efficient degradation of various emerging pollutants by wild type and evolved fungal DyP4 peroxidases
Source: PLoS One. 2022 Jan 13;17(1):e0262492. doi: 10.1371/journal.pone.0262492 (PMC8757903; doi:10.1371/journal.pone.0262492)
Supplement: Attachment — Submitted filename: PONE-D-21-27995 - Response to Reviewers - 16Dec2021.docx [file pone.0262492.s002.docx]

**PONE-D-21-27995**

Efficient degradation of various emerging pollutants by wild type and evolved fungal DyP4 peroxidases

**Response to Reviewers**

Reviewer #1: I have gone through the article "Efficient degradation of various emerging pollutants by wild type and evolved fungal DyP4 peroxidases" This is an interesting topic that is closely related to the scope of PLOS ONE. However, the manuscript should be improved in a number of following areas:

1- The introduction needs to be enhanced. More recent references are needed and some reference which is not related to this article should be avoided.

Response: Thank you for your comment. As requested, new recent references have been added (lines 46, 52, 67, 79, 82, 90). Some of the old references have also been avoided (lines 42, 46, 53). Unrelated references have also been removed.

2- It is recommended to discuss and compare the current study results with previously published studies in a TABLE form.

Response: Thank you very much for pointing this out. A table comparing previously published findings on 2-mercaptobenzothiazole degradation to the results of this study has been made and added to the manuscript.

3-It is suggested to write the equations and provide the graph of linear portion of absorbance Curve to calculate the Slope as the representation on line 146-147(May be as Supplementary Information).

Response: Thank you for your comment. We have included a supplemental figure that shows a representative data analysis, including linear regression and data fitting with the appropriate equations for determining the slope.

Reviewer #2: I have gone through the article "Efficient degradation of various emerging pollutants by wild type and evolved fungal DyP4 peroxidases" This is an Interesting Topic that is closely related to the scope of PLOS ONE. However, minor revisions are suggested for following areas:

1-Introduction need to be improved by removing irrelevant information, old references and by the addition of some recent studies.

Response: Thank you very much for commenting on this. New recent references have been added. Some of the old references have also been avoided. Unrelated references have also been removed.

2- compare the results of your study with previous studies in a separate table (year of study, researcher, methodology, main findings)

Response: Thank you for your comment. A table comparing previously published findings on 2-mercaptobenzothiazole degradation to the results of this study has been made and added to the manuscript.

3-Please write the equations in equation form for a clear understanding

Response: Thank you very much for your comments. The equation representing % percentage remaining has been written in an equation form.

4-Conclusion is needed to be revised

5- Define how this study will be beneficial on commercial scale

Response: Thank you for this suggestion. As requested, the conclusion has been significantly expanded and now also includes discussion (pasted below) about the future application of this technology on commercial scale.

*“This finding is very significant as one of the major limitations of using peroxidases for bioremediation is their potential oxidation and denaturation by the H2O2 that is needed for their activity. Results presented here suggest that enzyme engineering approach that was used to generate H2O2-tolerant variants of DyP4 peroxidases (3F6 and 4D4) can generate more robust, stable, and powerful bioremediation agents. The obvious next step would be to scale-up enzyme-mediated remediation approaches by immobilizing cheaply produced recombinant enzymes (or their improved variants) onto solid supports to create bioreactors/columns and test them on real-life wastewater. We have recently shown that soybean peroxidase can be efficiently supported on photocatalytic supports to produce novel hybrid biocatalysts more potent than the enzyme or the photocatalysts ((Morsi et al., 2021).”*
